# Healthcare use by people who use illicit opioids (HUPIO): development of a cohort based on electronic primary care records in England

**DOI:** 10.12688/wellcomeopenres.16431.2

**Published:** 2021-05-05

**Authors:** Dan Lewer, Prianka Padmanathan, Muhammad Qummer ul Arfeen, Spiros Denaxas, Harriet Forbes, Arturo Gonzalez-Izquierdo, Matt Hickman

**Affiliations:** 1 Institute of Health Informatics, University College London, London, NW1 2DA, UK; 2 Institute of Epidemiology and Healthcare, University College London, London, WC1E 7HB, UK; 3 Population Health Sciences, Bristol Medical School, University of Bristol, Bristol, BS8 1UD, UK; 4 National Institute of Health Research Biomedical Research Centre, Bristol NHS Foundation Trust and the University of Bristol, Bristol, UK

**Keywords:** substance use disorders, drug dependence, opioid agonist therapy, illicit drugs, electronic health records

## Abstract

**Background: **People who use illicit opioids such as heroin have substantial health needs, but there are few longitudinal studies of general health and healthcare in this population. Most research to date has focused on a narrow set of outcomes, including overdoses and HIV or hepatitis infections. We developed and validated a cohort using UK primary care electronic health records (Clinical Practice Research Datalink GOLD and AURUM databases) to facilitate research into healthcare use by people who use illicit opioid use (HUPIO).

**Methods: **Participants are patients in England with primary care records indicating a history of illicit opioid use. We identified codes including prescriptions of opioid agonist therapies (methadone and buprenorphine) and clinical observations such as ‘heroin dependence’. We constructed a cohort of patients with at least one of these codes and aged 18-64 at cohort entry, with follow-up between January 1997 and March 2020. We validated the cohort by comparing patient characteristics and mortality rates to other cohorts of people who use illicit opioids, with different recruitment methods.

**Results:** Up to March 2020, the HUPIO cohort included 138,761 patients with a history of illicit opioid use. Demographic characteristics and all-cause mortality were similar to existing cohorts: 69% were male; the median age at index for patients in CPRD AURUM (the database with more included participants) was 35.3 (interquartile range 29.1-42.6); the average age of new cohort entrants increased over time; 76% had records indicating current tobacco smoking; patients disproportionately lived in deprived neighbourhoods; and all-cause mortality risk was 6.6 (95% CI 6.5-6.7) times the general population of England.

**Conclusions: **Primary care data offer new opportunities to study holistic health outcomes and healthcare of this population. The large sample enables investigation of rare outcomes, whilst the availability of linkage to external datasets allows investigation of hospital use, cancer treatment, and mortality.

## Introduction

Opioids are a class of controlled drugs that include illicit substances such as heroin, substitution therapies such as methadone and buprenorphine, and pain medication such as morphine and codeine. While these drugs have both therapeutic and recreational uses, compared with most psychoactive drugs there is a high risk of physical or psychological dependence ^1^. The Diagnostic and Statistical Manual of Mental Disorders describes mild, moderate or severe ‘opioid use disorders’ ^2^ and the International Classification of Diseases provides criteria for ‘harmful patterns of use of opioids’ and ‘opioid dependence’ ^3^.

The frequency of illicit opioid use is difficult to estimate, in part because people who use these drugs are poorly represented in traditional epidemiological surveys. One study suggests that 0.8% of people aged 15–64 in England are dependent on illicit opioids ^4^, corresponding to approximately 300,000 individuals. There have also been growing concerns about dependence on prescription opioids, although the scale of this problem remains unclear ^5, 6^.

The health harms associated with illicit opioids are well-known. Many cohort studies have found high mortality rates ^7^. Use of illicit opioids is directly associated with multiple health and social harms such as infections, accidents, homelessness, and imprisonment ^1^. Co-occurrence of tobacco smoking, poor nutrition, and poor access to healthcare mean that almost all causes of death are more common among people who use illicit opioids than the general population ^8^.

The main treatment for dependence on illicit opioids is opioid agonist therapy (OAT); a pharmacological treatment of long-acting opioids such as methadone or buprenorphine. In England, OAT is provided by specialist drug and alcohol services or GPs, depending on local commissioning arrangements. A large body of evidence demonstrates the effectiveness of OAT across outcomes including mortality, physical and mental health, and criminal activity ^9– 13^.

Despite extensive evidence regarding the risks of illicit opioids and the benefits of OAT, there are important unanswered research questions. Epidemiological research and health interventions have focused on outcomes perceived to be ‘drug-related’, such as overdoses and HIV or hepatitis infections. Meanwhile, there is limited research into engagement with primary care services, healthcare quality, and treatment options for non-communicable diseases and mental health problems ^14^. These are important areas of research because the population of people who use illicit opioids in England (as in many other countries) is ageing ^15^ and the majority of excess deaths are now caused by non-communicable diseases such as liver disease, chronic obstructive pulmonary disease, and cardiovascular disease ^16^.

To facilitate research in these areas, we aimed to develop and validate a phenotype that identifies people with a history of illicit opioid use in longitudinal UK primary care electronic health records (Clinical Practice Research Datalink GOLD and AURUM databases).

## Methods

### Study design

We developed and validated a cohort of people with a history of illicit opioid use, including cross-sectional analysis of participant characteristics at baseline, and a cohort analysis of all-cause mortality rates.

### Data sources

The Clinical Practice Research Datalink (CPRD) GOLD and AURUM are databases of anonymised electronic health records from primary care, including approximately 8% and 13% of the populations in the UK and England respectively ^17– 19^. Although the databases include similar clinical information, they differ in terms of data collection software, clinical classification system and geographical coverage. CPRD GOLD includes data from GP practices throughout the UK, while CPRD AURUM initially included England only, and more recently practices in Northern Ireland have been added. To maximise comparability we have restricted the cohort to patients registered in England, though the methods can be used to include patients in other parts of the UK.

### Entry and exit dates

We selected patients who were registered at participating GP practices between 1 January 1997 and 31 December 2018 for GOLD, and between 1 January 1997 and 31 March 2020 for AURUM. Cohort entry was defined as the latest of 1 January 1997, the first date when good quality data were available for that patient, and the date of the first code indicating illicit opioid use. Cohort exit was the earliest of the date when the patient stopped being observed (‘last collection date’) or participating in CPRD (the patient transferred out of a participating GP practice), or death as recorded in their primary care record. In addition to these criteria, we excluded patients who were aged under 18 or 65 or older at cohort entry.

### Selection of patients with a history of illicit opioid use

We focused on patients with a history of opioid use (rather than specifically current use) due to the typically long duration of opioid use ^20– 22^ and the likelihood that patients would not have regularly recorded opioid use. We therefore included patients with illicit opioid use recorded prior to the cohort entry date.

**Figure 1.**
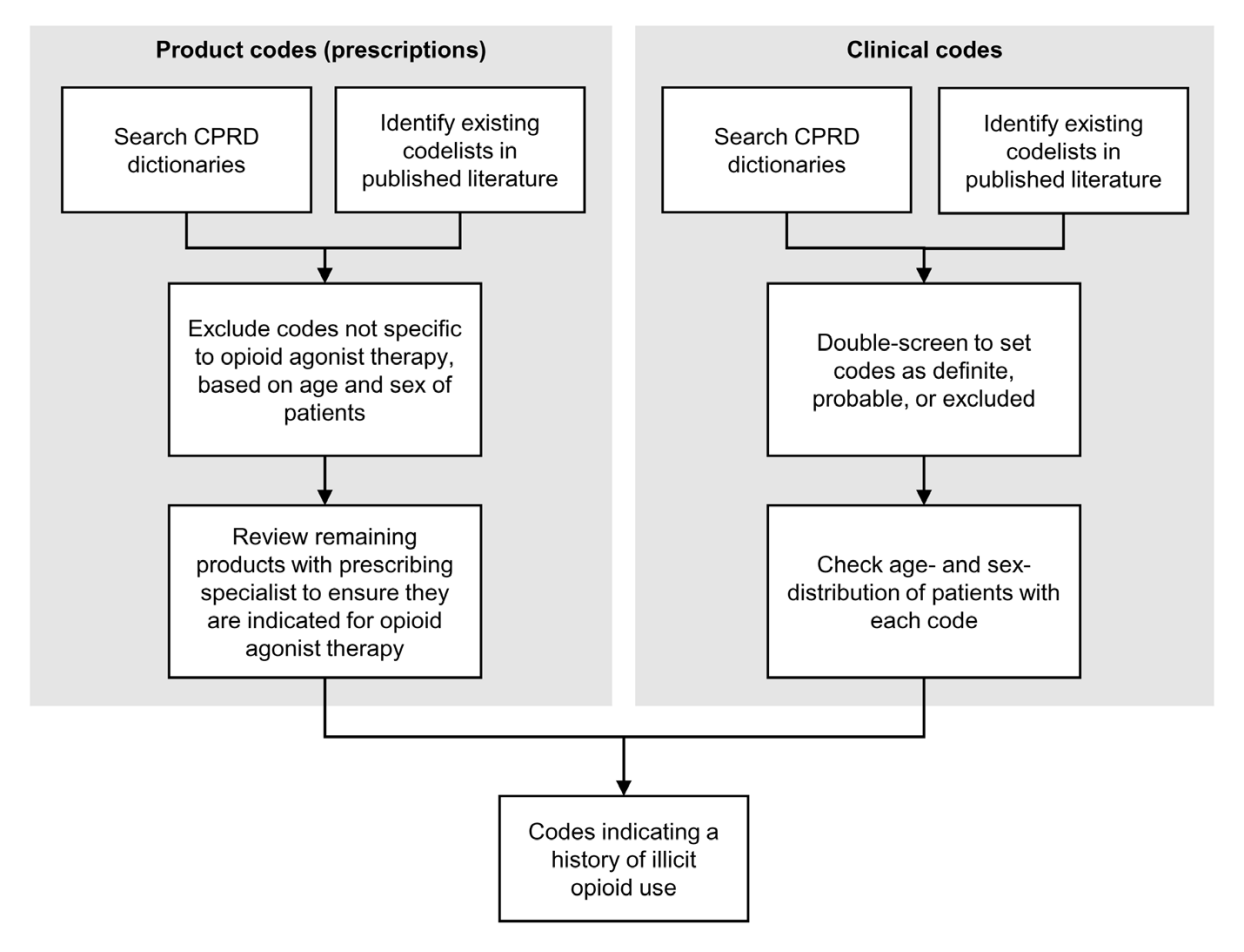
Selection of codes that indicate a history of illicit opioid use.

CPRD data include two main types of codes: product codes and clinical codes. Product codes indicate a prescription made in a primary care setting, whilst clinical codes indicate a diagnosis or other clinical observation (sometimes also a prescription). We selected patients by identifying product codes indicating a prescription of OAT and clinical codes indicating a history of illicit opioid use, such as ‘heroin dependence’ (see extended data for a full code list ^23^). We prioritised specificity over sensitivity, aiming to use codes that are only applied to the target population. Our process for selecting codes is summarised in Figure 1.

***Product codes.*** In the UK, treatment for opioid dependence involves the prescription of methadone or buprenorphine ^24^. However, these medications are also licensed for other indications including pain and palliative cough ^25, 26^. We therefore developed a method to identify medications that are specific to OAT.

We searched CPRD dictionaries to identify all methadone and buprenorphine product codes (full search terms are available as extended data ^23^). We found 175 codes in CPRD GOLD and 136 codes in CPRD AURUM. We compared these lists to an existing list of OAT medicines ^27^ and found no additional codes. We then followed a two-step process to identify products specific to OAT. First, we described the age- and sex-distribution of patients at the time of the first prescription. This showed two distinct groups: drugs mainly prescribed to younger men, and drugs mainly prescribed to older women (see extended data ^23^). Data from specialist drug treatment services shows that the population receiving OAT is three-quarters male and predominantly aged 18–64 ^28^. In contrast, the population prescribed opioids for pain relief is mainly older and female ^29^. We therefore excluded medications where more than half of patients were female, the lower quartile of age was younger than 18 years, or the upper quartile of age was older than 64, as these codes are unlikely to relate specifically to OAT. The majority of codes excluded were transdermal buprenorphine patches, which are not indicated for OAT ^26^. Second, a prescribing professional working in a community drug and alcohol service reviewed remaining products to check they are used for OAT.

***Clinical codes.*** CPRD GOLD and AURUM differ in the clinical coding system used. CPRD GOLD uses Read codes whilst AURUM uses SNOMED codes. We used keywords to search CPRD dictionaries to find Read and SNOMED clinical codes that may indicate illicit opioid use (methadone; buprenorphine; abus*; addict; dependen*; drug user; heroin; inject; misus*; opiate; opioid; overdose). Our search identified 1,098 Read codes and 1,800 SNOMED codes. Two authors (DL and PP) screened the codes for relevance, with conflicts resolved through discussion.

Where codes were likely to indicate illicit opioid use, but did not specifically mention opioids, we classified them as ‘probable’. For example, codes indicating injection of illicit drugs were classified as ‘probable’ because an estimated 94% of people who inject drugs in the UK use heroin ^30^. These codes have been excluded from our analyses to maximise specificity, but can be included in future research if greater sensitivity is needed.

Some clinical codes described prescriptions, tests or adverse reactions relating to methadone and buprenorphine. We excluded these where the indication was unclear.

After agreeing a list of codes, we checked the age- and sex-distribution of patients with these codes in the same way as we did with the product codes. A small number of codes were either prescribed to a majority of female patients or had an upper quartile of age older than 64. All of these codes represented dependence on medications prescribed for analgesia (for example ‘misuse of Codeine tablets’), which we classified as ‘probable’ and excluded from our analysis.

### External validation

We validated the HUPIO cohort by comparing it to other samples of people who use opioids. We anticipated the following characteristics: (a) the average age of patients entering the cohort would increase over time, as the cohort of people who use illicit opioids in England is ageing ^15^; (b) high prevalence of smoking, with a systematic review finding an average of 84% of people enrolled in addiction services currently smoke ^31^; and 70% of patients starting treatment for opioid dependence in England in 2018 recorded as current tobacco smokers ^28^. We reported the prevalence of current- and ex-smoking based on existing codelists for smoking histories ^32^; (c) disproportionate representation of patients living in more deprived areas, as illicit opioid use and opioid-related deaths are consistently associated with deprivation ^33, 34^; (d) higher mortality rates than the general population, as studies of mortality in this population consistently show very high mortality rates ^7^. We compared the standardised mortality ratios (SMR) for our cohort to those reported in existing studies of all-cause mortality in this population in England, identified by a brief literature search using Pubmed using the terms (opiate OR opioid OR heroin) AND (mortality OR death) without restrictions on language or publication date.

In addition to these characteristics, we reported the proportion of patients with recorded histories of homelessness, prison, and alcohol dependence, based on existing phenotypes ^35^ and searches of clinical codes. We expected these experiences to be common among people with a history of illicit opioid use ^36^. However, we did not know how consistently these experiences would be recorded in primary care, and therefore did not use these variables for validation purposes.

### Internal validation

We used hospital admissions where the patient had a diagnosis of ‘mental and behavioural disorders due to use of opioids’ (ICD10 code F11) to test the sensitivity of the primary care codelist. Among all patients in CPRD with linked Hospital Episode Statistics data, we requested dates of hospital admissions where F11 was recorded in any diagnostic position. We then reported the proportion of these patients who had a primary care code indicating a history of illicit opioid use based on the HUPIO codelist, and whether the primary care code was before the first hospital admission, in the 30 days after the first admission, or more than 30 days after admission. We compared the timing of these events because information may be recorded in primary care databases following receipt of hospital discharge summaries.

### Statistical analysis (estimation of mortality rates and ratios)

We calculated mortality rates and standardised ratios for the subset of patients with linked ONS mortality data (further detailed about the linkage process are available in CPRD documentation) ^18, 19^. We requested mortality data for these patients, with a final date of follow-up of 1 May 2019. To minimise bias due to delayed death registration, which is likely to occur disproportionately for people who use illicit opioids due to the involvement of coroners in deaths due to drug poisoning, we stopped follow-up six months before this date (i.e. 30 October 2018). To calculate the standardised mortality ratio, we: (a) calculated the duration of follow-up in the HUPIO cohort, stratified by sex, single-year-of-age, and calendar year. We accounted for aging by expanding follow-up for each participant into days, and summarising the number of days by sex, single-year-of-age and calendar year; (b) applying mortality rates in the general population of England ^37^ to these strata to calculate a number of expected deaths; (c) dividing the number of observed deaths by expected deaths and calculating 95% confidence intervals using the exact Poisson method.

All data manipulation and analysis was conducted using R version 3.6.2 ^38^.

### Patient and public involvement

People who use illicit opioids were involved in discussions about the need for research into health and healthcare for this population.

## Results

### Cohort size and characteristics

**Figure 2.**
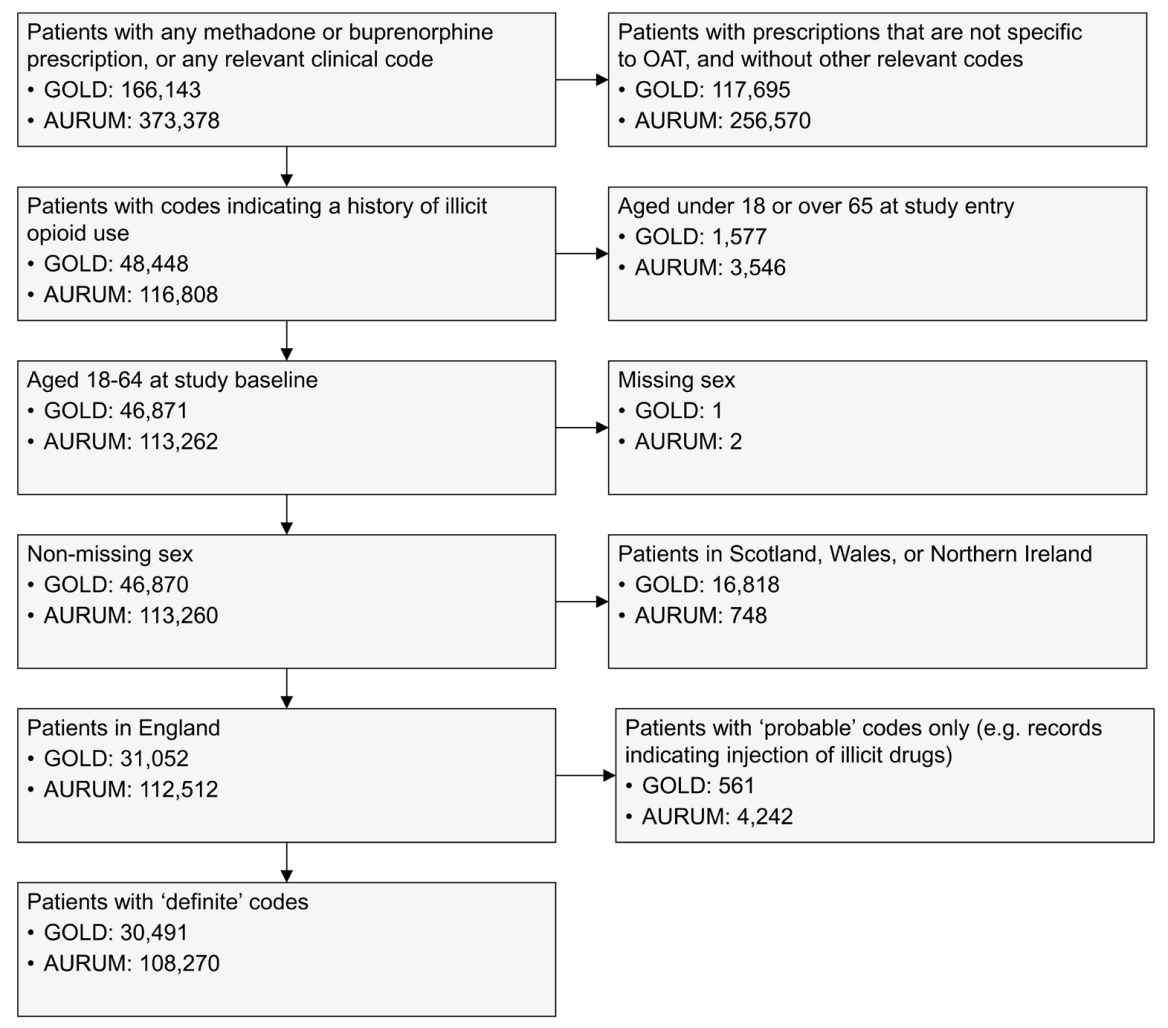
Flow-chart of the number of participants in the cohort. OAT - opioid agonist therapy.

After exclusions, the HUPIO cohort included 30,491 individuals from GOLD and 108,270 individuals from AURUM. The derivation of the cohort is shown in Figure 2. The number of participants under active follow-up in GOLD increased over time to a maximum of 11,384 in March 2010 and then decreased as GP practices stopped contributing data; and in AURUM increased over time to a maximum of 44,935 in October 2018.

**Figure 3.**
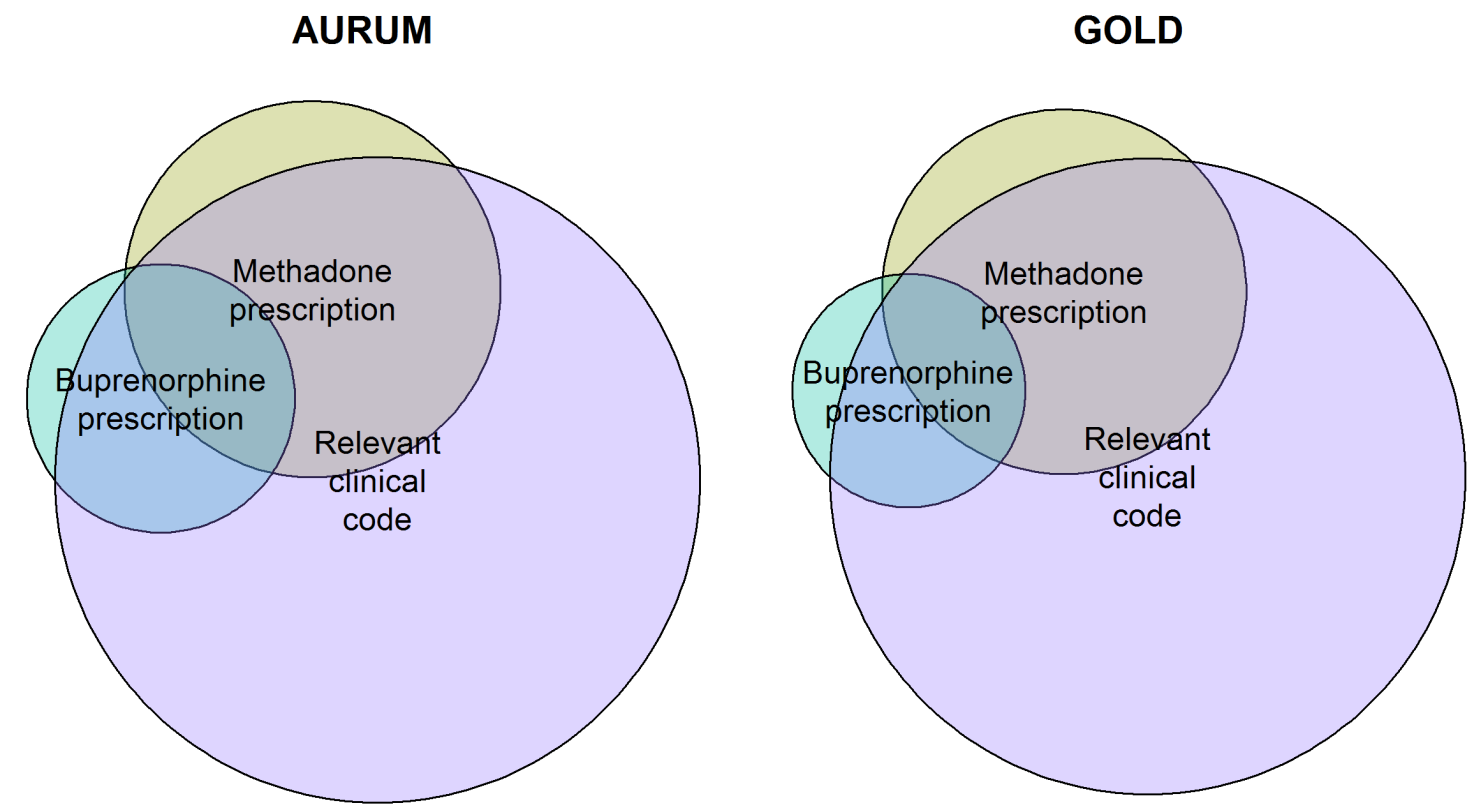
Venn diagram showing the overlap between individuals with a history of opioid agonist prescriptions in primary care and clinical codes indicating non-therapeutic opioid use. Note that there are fewer patients in GOLD and this is not reflected in the relative sizes of the circles.

The majority of patients in both databases had a relevant clinical code and no OAT prescription, while the majority of patients with an OAT prescription also had a relevant clinical code ( Figure 3).

The median age at baseline was 33.5 in GOLD and 35.3 in AURUM. The distribution of age groups was similar to that of patients entering treatment for opioid dependence in England (see extended data ^23^). We observed a linear increase in the mean age of patients entering our cohort, parallel and three years older than the mean age of patients entering treatment for opioid dependence in England (see extended data ^23^). When stratified by date, the mean age of patients entering the cohort was similar for GOLD and AURUM. The older average age in AURUM is therefore explained by patients entering the cohort at later dates than in GOLD. In both GOLD and AURUM, 69% of patients were male; similar to 72% of patients in opioid agonist treatment in England in 2018 ^28^, and 72% of participants in the Unlinked Anonymous Monitoring Survey of People who Inject Drugs ^39^. There was a clear association between deprivation and a history of opioid use, with over 40% of patients living in the most deprived quintile of neighbourhoods.

In both databases, approximately three-quarters of patients were current smokers (at the most recent record of smoking) and a further 10% were ex-smokers. Characteristics of patients are shown in Table 1.

**Table 1.** Characteristics of patients in primary care with a history of illicit opioid use.

Variable	Level	GOLD: 1997–2018 N (%)	AURUM: 1997–2020 N (%)
Total		30,491	108,270
Follow-up, years	Median (IQR) *	3.5 (1.8-7.0)	3.8 (1.9-8.1)
Age at index	18–29	10,585 (34.7)	30,444 (28.1)
	30–39	11,727 (38.5)	42,251 (39.0)
	40–49	5,866 (19.2)	24,955 (23.0)
	50–64	2,313 (7.6)	10,620 (9.8)
	Median (IQR)	33.5 (27.6-40.7)	35.3 (29.1-42.6)
Sex	Male	21,141 (69.3)	75,065 (69.3)
	Female	9,350 (30.7)	33,205 (30.7)
Region	East Midlands	1,705 (5.6)	1,846 (1.7)
	East of England	2,735 (9.0)	2,604 (2.4)
	London	3,016 (9.9)	16,876 (15.6)
	North East	911 (3.0)	5,080 (4.7)
	North West	6,128 (20.1)	23,236 (21.5)
	South East	5,675 (18.6)	15,649 (14.5)
	South West	4,407 (14.5)	20,208 (18.7)
	West Midlands	4,071 (13.4)	18,241 (16.8)
	Yorkshire & The Humber	1,843 (6.0)	4,086 (3.8)
	Missing	0 (0.0)	444 (0.4)
Smoking	Current	23,647 (77.6)	81,968 (75.7)
	Ex-smoker	2,764 (9.1)	11,016 (10.2)
	Never	1,613 (5.3)	6,689 (6.2)
	No records	2,467 (8.1)	8,597 (7.9)
Quintile of index of multiple deprivation (patient-level) **	1 (least deprived)	2,023 (6.6%)	
	2	3,357 (11.0%)	
	3	4,846 (15.9%)	
	4	7,383 (24.2%)	
	5 (most deprived)	12,870 (42.2%)	
	Missing	12 (<0.1%)	
Homelessness		1,143 (3.7)	6,219 (5.7)
Prison		2,101 (6.9)	8,397 (7.8)
Alcohol dependence		4,499 (14.8)	22,150 (20.5)

^*^ Follow-up time in this table refers to time that patients are observed within CPRD and primary care records are available. If external databases such as Hospital Episode Statistics or ONS mortality are use, longer follow-up is available. ^**^ Index of Multiple Deprivation was not available for patients in CPRD AURUM at the time of publication

### Mortality rates and ratios

In CPRD AURUM, linkage to ONS mortality records was conducted for 75,807/108,270 patients (70%), and in CPRD GOLD, linkage was conducted for 23,241/30,491 patients (76%). Mortality rates were similar in the two databases. During a combined 910,567 patient-years of follow-up, there were 12,404 deaths (crude mortality rate of 13.6 deaths per 1,000 person-years). Given age- and sex-specific mortality rates in the general population of England, we expected 1,872 deaths, giving an SMR of 6.6 (95% CI 6.5-6.7). Table 2 provides a summary of follow-up time, deaths, and mortality rates, stratified by database, age and sex. We identified two studies that also reported SMRs in populations with a history of illicit opioid use in England. The first identified 198,247 opiate users from national drug treatment and criminal justice databases between 2005 and 2009 ^8^ and used linkage to national mortality records to estimate an SMR of 5.7 (95% CI 5.5-5.9). The second identified 6,683 people entering treatment for heroin dependence in South London between 2006 and 2019 ^16^, again using linkage to national mortality records to calculate an SMR of 6.6 (95% CI 6.1-7.1). In both studies, as in our cohort, the crude mortality rate was higher for men, while the SMR was higher for women.

**Table 2.** All-cause mortality rates and standardised mortality ratios for patients in primary care in England with a history of illicit opioid use.

Database	Sex	Age group	Number at baseline	Follow-up (years)	Observed deaths	Expected deaths	CMR (95% CI)	SMR (95% CI)
AURUM	Female	18–29	7,176	28,308	105	8.4	3.71 (3.03-4.49)	12.56 (10.28-15.21)
		30–39	8,729	78,140	613	44.7	7.84 (7.24-8.49)	13.71 (12.65-14.84)
		40–49	4,840	60,461	788	79.4	13.03 (12.14-13.98)	9.92 (9.24-10.64)
		50–64	2,490	31,612	771	116.1	24.39 (22.70-26.17)	6.64 (6.18-7.13)
		65+	-	4,672	176	59.4	37.67 (32.31-43.67)	2.97 (2.54-3.44)
		All ages	23,235	203,193	2,453	308.0	12.07 (11.60-12.56)	7.97 (7.65-8.29)
	Male	18–29	13,305	46,228	358	33.0	7.74 (6.96-8.59)	10.85 (9.76-12.04)
		30–39	21,409	177,614	1,552	190.0	8.74 (8.31-9.18)	8.17 (7.77-8.58)
		40–49	12,899	166,047	2,472	349.2	14.89 (14.31-15.49)	7.08 (6.80-7.36)
		50–64	4,959	73,266	2,107	388.2	28.76 (27.54-30.01)	5.43 (5.20-5.66)
		65+	-	6,453	277	119.1	42.92 (38.02-48.29)	2.33 (2.06-2.62)
		All ages	52,572	469,608	6,766	1079.5	14.41 (14.07-14.76)	6.27 (6.12-6.42)
	Both	18–29	20,481	74,536	463	41.3	6.21 (5.66-6.80)	11.20 (10.20-12.27)
		30–39	30,138	255,754	2,165	234.7	8.47 (8.11-8.83)	9.22 (8.84-9.62)
		40–49	17,739	226,507	3,260	428.6	14.39 (13.90-14.90)	7.61 (7.35-7.87)
		50–64	7,449	104,879	2,878	504.3	27.44 (26.45-28.46)	5.71 (5.50-5.92)
		65+	-	11,125	453	178.4	40.72 (37.06-44.65)	2.54 (2.31-2.78)
		All ages	75,807	672,801	9,219	1387.5	13.70 (13.42-13.99)	6.64 (6.51-6.78)
GOLD	Female	18–29	2,455	10,709	34	3.2	3.18 (2.20-4.44)	10.71 (7.42-14.97)
		30–39	2,612	28,101	198	16.1	7.05 (6.10-8.10)	12.33 (10.68-14.18)
		40–49	1,374	21,245	278	27.9	13.09 (11.59-14.72)	9.98 (8.84-11.22)
		50–64	721	11,303	238	42.1	21.06 (18.47-23.91)	5.65 (4.95-6.41)
		65+	-	1,977	59	25.8	29.85 (22.72-38.50)	2.29 (1.74-2.95)
		All ages	7,162	73,334	807	115.0	11.00 (10.26-11.79)	7.02 (6.54-7.52)
	Male	18–29	4,768	17,955	147	12.9	8.19 (6.92-9.62)	11.38 (9.62-13.38)
		30–39	6,515	64,132	599	68.3	9.34 (8.61-10.12)	8.77 (8.08-9.50)
		40–49	3,525	55,817	830	116.9	14.87 (13.88-15.92)	7.10 (6.62-7.60)
		50–64	1,271	24,227	691	129.0	28.52 (26.43-30.73)	5.35 (4.96-5.77)
		65+	-	2,300	111	42.7	48.27 (39.71-58.12)	2.60 (2.14-3.13)
		All ages	16,079	164,431	2,378	369.9	14.46 (13.89-15.06)	6.43 (6.17-6.69)
	Both	18–29	7,223	28,664	181	16.1	6.31 (5.43-7.30)	11.25 (9.67-13.02)
		30–39	9,127	92,233	797	84.4	8.64 (8.05-9.26)	9.45 (8.80-10.13)
		40–49	4,899	77,062	1,108	144.8	14.38 (13.54-15.25)	7.65 (7.21-8.12)
		50–64	1,992	35,530	929	171.2	26.15 (24.49-27.88)	5.43 (5.08-5.79)
		65+	-	4,276	170	68.5	39.75 (34.00-46.20)	2.48 (2.12-2.88)
		All ages	23,241	237,766	3,185	484.9	13.40 (12.93-13.87)	6.57 (6.34-6.80)
Total			99,048	910,567	12,404	1872.4	13.62 (13.38-13.86)	6.62 (6.51-6.74)

CMR = crude mortality rate (per 1000 person-years) SMR = standardised mortality ratio (compared to the general population of England)

### Internal validation

Among patients hospitalised with a diagnosis of ‘mental and behavioural disorders due to use of opioids’, 89% of patients in GOLD and 88% of patients in AURUM also have a record in primary care data indicating a history of illicit opioid use. 72% and 67% of hospitalised patients respectively have the first relevant primary care code prior to hospitalisation, 2% and 3% have the primary code in the 30 days after hospitalisation, and 15% and 18% have the code more than 30 days after hospitalisation. A table of this information is provided in extended data.

## Discussion

We developed and validated an electronic healthcare record phenotype that identified approximately 139,000 patients with a history illicit opioid use registered at primary care practices in England. Patient characteristics (age, sex, smoking history, and deprivation) and mortality rates were comparable to other samples of this population.

### Strengths and limitations

To our knowledge, this is the first study to develop a method for identifying people with a history of illicit opioid use within primary care records. Earlier studies have focused specifically on people prescribed opioids ^40, 41^, general illicit drug use or dependence ^42, 43^, and people prescribed OAT ^28, 44^. The latter is a limited subset of this population, particularly given that OAT in England is not always prescribed by GPs. These studies have included patients prescribed any methadone or buprenorphine product and excluded those with doses suggesting indications other than OAT (such as pain or palliative cough). Yet over 70% of daily doses for these medications are missing from CPRD ^45^, and therefore require imputation or exclusion. The method developed in this study avoids the need for imputation by using products that are specific to OAT. It is possible that we excluded some medicines that are used for OAT in addition to other indications, though few patients with prescriptions of excluded methadone or buprenorphine products had other codes indicating a history of illicit opioid use (see extended data ^23^).

For individuals prescribed OAT in primary care, CPRD includes details of these prescriptions. However, in England, many individuals are prescribed OAT in other settings and information about these prescriptions is not available. The main alternative source of national data on people prescribed OAT in England is the National Drug Treatment Monitoring System, which provides data on people in specialist community drug and alcohol treatment services ^28^. Although the population using these services is likely to overlap with those in primary care, there may also be important differences. For example, people accessing only drug and alcohol services may have more complex drug treatment needs, whilst those only in primary care may have greater physical comorbidity.

The main strength of CPRD in relation to other research datasets for this population is that it offers unique insights into primary healthcare. It can be linked to external datasets to obtain information on care in hospitals, cancer services, and mental health services, as well as causes of mortality. Algorithms have also been developed to facilitate identification of particular aspects of healthcare such as pregnancy ^46^, or health outcomes such as cardiovascular disease ^47^.

There are also limitations in the data, particularly because all data are derived from routine healthcare records. For example, in CPRD there is no systematic recording of the type and frequency of drug use, and the degree of drug dependence. The data on smoking presented in this article suggests that some characteristics of this population are well-captured by GPs, as fewer than 10% had no records and the prevalence of smoking is comparable to that found in other studies. Other characteristics may be less well-captured, for example fewer-than-expected patients had records of homelessness or prison.

Depending on the research question, selection biases are likely to be important. To be included in the CPRD sample, individuals need to be registered with a GP, attend an appointment, and disclose their drug use. At present, we do not know what proportion of this population is registered with a GP. In one study of homeless people who inject drugs in London; a subgroup likely to have relatively high barriers to GP registration, 70% provided GP details ^48^, suggesting that a large proportion of this population is registered. However, disclosure of drug use is likely to differ. Groups more likely to disclose drug use may include those prescribed OAT (either in primary care or specialist drug and alcohol services), and those who are more unwell and therefore have more GP appointments. This latter factor may lead to an overestimation in differences in morbidity and mortality when comparing people with a history of illicit opioid use to the general population. Qualitative research has found both practice-level and individual-level barriers to disclosing and recording illicit drug use ^49^. In particular, patients and GPs who feel more stigma towards illicit drug use may be less likely to discuss the issue. Our internal validation suggested that approximately 90% of patients with opioid use recorded in hospitals also have illicit opioid use recorded in primary care, and the timings of these records suggest they are independent. This supports good sensitivity of the primary care codelist for patients who are registered at a GP practice. It does not provide evidence of sensitivity at a population level, because some individuals are not registered at a GP practice. Consideration of selection biases is important when interpreting analyses using this data and designing sensitivity analyses. Where possible, triangulation with other sources, such as the National Drug Treatment Monitoring System ^28^, can improve confidence in findings.

The data presented here covers a long period of time (1997–2020). Changes happened in both the population and in health services. For example, investment in opioid agonist therapy has changed; while the average age of the population and the prevalence of long-term conditions have increased. This means that selection biases are likely to change over follow-up. In some cases, it may help to restrict analysis to a shorter time-period, or stratify by time-period.

### Implications for future research

To date, research into people who use illicit opioids has focused mainly on a narrow range of outcomes such as blood borne viruses and overdoses. The average age of people with a history of illicit opioid use is increasing, and consequently the importance of chronic health conditions is also increasing ^16^. Key areas for future research include the epidemiology of these health issues, assessing the risks and benefits of existing interventions such as OAT in terms of a broader range of health outcomes, and understanding utilisation and quality of general healthcare for this population.

## Conclusion

People with a history of illicit opioid use have substantial unmet health needs. Yet to date, large-scale longitudinal studies of healthcare and holistic health outcomes in this population have been limited. We developed and validated a method of identifying people who have a history of illicit opioid use in primary care data to facilitate further research and support improvements in healthcare.

## Further details

### Ethics and approvals

The study was approved by the MHRA (UK) Independent Scientific Advisory Committee and 19_142R, under Section 251 (NHS Social Care Act 2006). This study is based in part on data from the Clinical Practice Research Datalink obtained under license from the UK Medicines and Healthcare products Regulatory Agency. The data are provided by patients and collected by the NHS as part of their care and support. The interpretation and conclusions contained in this study are those of the authors alone.

## Data availability

### Underlying data

Researchers can study the HUPIO cohort by applying to the CPRD Independent Scientific Advisory Committee (ISAC). Approval is required if access to anonymised patient level data is being requested for research purposes.

Details of the application process and conditions of access are provided by CPRD at https://www.cprd.com/Data-access.

### Extended data

UCL Discovery: Healthcare use by people who use illicit opioids (HUPIO): development of a cohort based on electronic primary care records in England (extended data). https://doi.org/10.5522/04/13253906 ^23^

This data file includes:

1. Search terms used to identify codes that may represent a history of illicit opioid use
2. Codelist for identifying people with a history of illicit opioid use
3. Age- and sex-distribution of patients by product and clinical codes
4. Number of patients currently in the cohort
5. Age of patients at cohort entry
6. Internal validation based on hospital admissions for opioid dependence

The phenotype identifying patients with a history of illicit opioid use can also be found on the CALIBER website at https://portal.caliberresearch.org/phenotypes/lewer-hupio-mzxe2uzxdzvybsabtjbhrk. Researchers who are accessing CPRD via the CALIBER platform can use the phenotype directly, while other researchers can download the codelist for their own use.

Data are available under the terms of the Creative Commons Attribution 4.0 International license (CC-BY 4.0).
