# Supplementary material for: Healthcare use by people who use illicit opioids (HUPIO): development of a cohort based on electronic primary care records in England
Source: Wellcome Open Res. 2021 May 5;5:282. Originally published 2020 Dec 3. [Version 2] doi: 10.12688/wellcomeopenres.16431.2 (PMC7901498; doi:10.12688/wellcomeopenres.16431.2)
Supplement: Peer review history

# Peer review: Reviewer response for version 2

**DOI:** 10.21956/wellcomeopenres.18411.r43677

**Published:** 2021-05-05

**Authors:** Sally McManus

**Affiliations:** 1 School of Health Sciences, City, University of London, London, UK; 2 Violence and Society Centre, City, University of London, London, UK; 3 National Centre for Social Research, London, UK

Excellent revisions, no further comments.

Is the work clearly and accurately presented and does it cite the current literature?

Yes

If applicable, is the statistical analysis and its interpretation appropriate?

Yes

Are all the source data underlying the results available to ensure full reproducibility?

Yes

Is the study design appropriate and is the work technically sound?

Yes

Are the conclusions drawn adequately supported by the results?

Yes

Are sufficient details of methods and analysis provided to allow replication by others?

Yes

Reviewer Expertise:

Population mental health, surveys, cohort studies, inequalities, social determinants

I confirm that I have read this submission and believe that I have an appropriate level of expertise to confirm that it is of an acceptable scientific standard.

# Peer review: Reviewer response for version 1

**DOI:** 10.21956/wellcomeopenres.18081.r42593

**Published:** 2021-02-22

**Authors:** Sally McManus

**Affiliations:** 1 School of Health Sciences, City, University of London, London, UK; 2 Violence and Society Centre, City, University of London, London, UK; 3 National Centre for Social Research, London, UK

This important methodological study sets out an approach for identifying a cohort of primary care patients in England with a history of illicit opioid use. Consistent with good Open Science principles, codes and algorithms are provided for transparency, to provide detail for future publications based on the cohort identified, and to enable other researchers to apply the same or an adapted approach in their own work with the CPRD, AURUM or related databases. The combined cohort produces an extremely large resource (over 135,000 people), and the longitudinal nature of the data will allow for progression and change to be tracked at the patient-level.

 Key external validation work is presented, confirming the cohort profile is consistent with other profiles and with expectations. It would be useful for future validation work to address what proportion of people with a history of illicit opioid use have this noted in their primary care records, and whether biases exist in whether this is a. reported by patients, and b. recorded by practitioners. For example, recognition may be more likely in those who use primary healthcare more, feel more able to disclose illicit behaviour, and for whom opioid use is having greater health consequences. Practitioners can be reluctant to record drug use, and there is useful qualitative research that could provide useful context on such biases (e.g. Davies-Kershaw ^1^).

 This will be a powerful and pragmatic tool for examining a wider than previously considered range of health outcomes for a cohort of people with a history of illicit opioid use, and their longer term progression.

 Minor:

- Spell out IQR acronym in abstract.
- Table 1 – if possible, would be good to have a column showing the profile for the total population and/or patients without a history of illicit opioid use.
- Table 2 – should the word ‘total’ be removed from rows 6 and 14, column 3?

Is the work clearly and accurately presented and does it cite the current literature?

Yes

If applicable, is the statistical analysis and its interpretation appropriate?

Yes

Are all the source data underlying the results available to ensure full reproducibility?

Yes

Is the study design appropriate and is the work technically sound?

Yes

Are the conclusions drawn adequately supported by the results?

Yes

Are sufficient details of methods and analysis provided to allow replication by others?

Yes

Reviewer Expertise:

Population mental health, surveys, cohort studies, inequalities, social determinants

I confirm that I have read this submission and believe that I have an appropriate level of expertise to confirm that it is of an acceptable scientific standard.

## References

[1] : Factors influencing recording of drug misuse in primary care: a qualitative study of GPs in England. *Br J Gen Pract*.68(669) : 10.3399/bjgp18X695309 e234-e244 10.3399/bjgp18X695309 29483076PMC5863677

# Response

**Published:** 2021-04-20

**Authors:** Dan Lewer

**Affiliations:** University College London, UK

Many thanks for reviewing our article. We have updated the article following your feedback. In addition, we have some specific responses to your comments and questions:

 Comment: It would be useful for future validation work to address what proportion of people with a history of illicit opioid use have this noted in their primary care records, and whether biases exist in whether this is a. reported by patients, and b. recorded by practitioners. For example, recognition may be more likely in those who use primary healthcare more, feel more able to disclose illicit behaviour, and for whom opioid use is having greater health consequences. Practitioners can be reluctant to record drug use, and there is useful qualitative research that could provide useful context on such biases (e.g. Davies-Kershaw).

 Response: We agree this is central to understanding the cohort. We have added some discussion about the sensitivity of the codelist (two paragraphs above the subheading 'Implications for future research'), including a reference to the article by Davies-Kershaw (thank you for highlighting this). We have also conducted an additional internal validation exercise. This involved selecting all patients in CPRD who had a diagnosis of ICD-10 F11 ('mental and behavioural disorders due to opioid use') and reporting how many appeared in the HUPIO cohort, which is based on primary care records only. This suggested good sensitivity, with approximately 90% of patients identified in hospital also appearing in HUPIO.

# Peer review: Reviewer response for version 1

**DOI:** 10.21956/wellcomeopenres.18081.r41740

**Published:** 2020-12-21

**Authors:** Dhanya Nambiar

**Affiliations:** 1 Turning Point, Melbourne, Australia; 2 Monash University, Melbourne, Australia

Summary:

 This paper described a method to establish a cohort of people with a history of opioid use in England using primary care electronic records from 1997 to 2020. Records were searched based on algorithm of primary care notes on opioid use and OAT. At validation, the cohort were found to be comparable to other cohorts of opioid users in the region by age, gender, tobacco use and mortality. The limitations of identifying opioid users from primary care records included people with more physical conditions, who were more connected to primary care and who more likely to disclose their drug use.

 Overall, the manuscript is well written and presented, and demonstrates good scientific methods and discussion. I have a few comments on the content and some questions for consideration:

 Abstract: 

- Background: Is there a typo in "people who use illicit opioid use" ?

Entry and Exit Dates:

- Over such a long recruitment period, are participants comparable in terms of case ascertainment ie. do you expect that identifying opioid use has become better or worse over the period? And have there been any changes in the healthcare system such as access to OAT or uptake of primary care over the recruitment period? 
- Clinical codes: "...Read and ENOMED clinical codes that may illicit opioid use..." - is there a word missing?
- External validation: Are all deaths reliably linked to both GOLD and AURUM records? 
- Results - cohort size and characteristics

In Table 1, considering the length of the study period, do you know why the median follow up period was relatively short at under 4 years? Do patients frequently transfer out of GP practices, or is this specific to this cohort?

- Are participants uniquely identifiable across different participating GP practices over the study period? In future, it would be interesting to compare the frequency at which they present to primary and other healthcare services. This may speak to some of the current limitations such as I) how representative the cohort is to other international opioid using populations in terms of health service use, and ii) determine if there are subgroups within the cohort with distinct patterns of primary care use (that may have other distinct characteristics such as drug use patterns, homelessness, deprivation, etc). 

Is the work clearly and accurately presented and does it cite the current literature?

Yes

If applicable, is the statistical analysis and its interpretation appropriate?

Yes

Are all the source data underlying the results available to ensure full reproducibility?

Yes

Is the study design appropriate and is the work technically sound?

Yes

Are the conclusions drawn adequately supported by the results?

Yes

Are sufficient details of methods and analysis provided to allow replication by others?

Yes

Reviewer Expertise:

Health service utilisation, drug use, opioids, cohort studies, mortality

I confirm that I have read this submission and believe that I have an appropriate level of expertise to confirm that it is of an acceptable scientific standard.

# Response

**Published:** 2021-04-20

**Authors:** Dan Lewer

**Affiliations:** University College London, UK

Many thanks for reviewing our article. We have updated the article following your feedback. In addition, we have some specific responses to your comments and questions:

 Comment: Over such a long recruitment period, are participants comparable in terms of case ascertainment ie. do you expect that identifying opioid use has become better or worse over the period? And have there been any changes in the healthcare system such as access to OAT or uptake of primary care over the recruitment period?

 Response: Yes, there have been changes in both the population and healthcare system that mean case ascertainment is likely to change over time. Two important examples are (1) decreasing investment in OAT since 2010, which may reduce case ascertainment because patients have less overall contact with health services (though OAT is generally delivered in specialist community settings rather than by GPs); (2) the increasing age of the population greater health needs - which may improve case ascertainment as people have a greater need to see their GP. Restricting analyses to limited time periods (or stratifying by time period) may help - which we have now recommended in the paragraph above 'implications for future research'.

 Comment: Are all deaths reliably linked to both GOLD and AURUM records?

 Response: The first version of this article used death data in primary care records. Since then, we have received linked mortality data from the Office for National Statistics for the HUPIO cohort. The mortality rates and ratios in the revised paper use this data. While there are undoubtedly errors in the linkage process, it is generally considered to be high quality and miss few deaths. The linkage uses NHS numbers, a unique identifier that is assigned to everyone registered at a GP practice, in combination with other identifiers. Further information about the linkage methodology is available here: Padmanabhan S, Carty L, Cameron E, Ghosh RE, Williams R, Strongman H. Approach to record linkage of primary care data from Clinical Practice Research Datalink to other health-related patient data: overview and implications. Eur J Epidemiol. 2019;34(1):91-99. doi:10.1007/s10654-018-0442-4

 Comment: In Table 1, considering the length of the study period, do you know why the median follow up period was relatively short at under 4 years? Do patients frequently transfer out of GP practices, or is this specific to this cohort?

 Response: There are two reasons for this. First, GP practices are continually joining and exiting CPRD. The pool of practices participating in CPRD GOLD is shrinking, and when a practice leaves the database then follow-up for the patient ends. Likewise, the pool of practices for CPRD AURUM is growing and many practices joined later than 1997. Second, this is a mobile population and patients do frequently transfer out of GP practices. We have added a note to the table to clarify that these values are the follow-up duration for primary care data only, and longer follow-up is available where linked data are used (such as Hospital Episode Statistics or Office for National Statistics mortality records). In later analyses we will used matched cohorts of patients who do not have a history of illicit opioid use, for comparison. In these cohorts, follow-up is typically a bit longer because the general population is less mobile than people who use illicit opioids.

 Comment: Are participants uniquely identifiable across different participating GP practices over the study period? In future, it would be interesting to compare the frequency at which they present to primary and other healthcare services. This may speak to some of the current limitations such as I) how representative the cohort is to other international opioid using populations in terms of health service use, and ii) determine if there are subgroups within the cohort with distinct patterns of primary care use (that may have other distinct characteristics such as drug use patterns, homelessness, deprivation, etc).

 Response: No, unfortunately participants are not uniquely identifiable across different participanting GP practices. It is possible that some patients move from one participating practice to another, and therefore appear in the data twice. This is thought to be sufficiently rare in CPRD that each "patient" (in fact a patient-registration episode) can reasonably be considered unique. We agree that it will be interesting to analyse healthcare frequency. CPRD provides linkage to hospital data, and this will allow comparison with existing studies of hospital admissions in patients recruited via specialist OAT clinics.
